# Diffusion tensor imaging in patients with idiopathic normal pressure hydrocephalus

**DOI:** 10.1186/2045-8118-12-S1-P42

**Published:** 2015-09-18

**Authors:** Tomas Radovnicky, Daniel Adamek, Milous Derner, Martin Sames

**Affiliations:** 1 Department of Neurosurgery, Masaryk Hospital, Usti nad Labem, Czech Republic; 2 Department of Radiology, Masaryk Hospital, Usti nad Labem, Czech Republic

## Introduction

Idiopathic normal pressure hydrocephalus (iNPH) is well known as a treatable syndrome affecting an elderly people. There is a lack of specific radiological features, which can identify the iNPH patient. Diffusion tensor imaging (DTI) is a MRI technique for evaluation of the microstructural white matter characteristics, which can be affected by iNPH. Aim of this study was to find specific DTI sign for iNPH diagnosis.

## Methods

This study was designed as a prospective, blinded, single institution study. We analysed MRI of 27 patients before the shunt surgery and 1 year after. DTI parameters (fractional anisotropy – FA, mean diffusivity – MD) were examined by 1, 5 T MRI in previously defined areas – the anterior and posterior limb of the internal capsule, the corpus callosum. iNPH patients were identified by clinical examination, dilatation of ventricles on MRI defined by Evans` ratio > 0, 30 and positive tap test and/or lumbar infusion test. Patient outcome was measured by iNPH grading scale one year after surgery. As a control group we examined 24 age-matched healthy controls. DTI values were statistically analysed.

## Results

FA in the posterior limb of the internal capsule was significantly higher in iNPH patients compared to the healthy controls (0, 70 vs. 0, 58, p < 0, 05).

The analysis of FA and MD values before and after the shunt surgery shows the significant decrease of FA in the posterior limb of the internal capsule (mean 0, 70 before and 0, 63 after the surgery, p < 0, 05). But the postoperative FA in the posterior limb of the internal capsule was still higher than in the healthy control group. No significant change in other areas was identified.

In the group of 27 iNPH patients were 4 shunt non-responders (14, 8%). No significant difference between FA and MD values in responders and non-responders was found.

## Conclusions

Fractional anisotropy value in the posterior limb of the internal capsule was significantly higher in iNPH patients compared to the healthy control group. This value significantly decreased after the surgery. But it failed in the differentiation of patients with response to the shunt surgery from the non-responders. Higher FA in the posterior limb of the internal capsule is supportive for iNPH diagnosis, but it shouldn`t be used as a single predictor.

Supported by IGA NT14448-3/2013.

